# Radiation-associated cardiovascular risks for future deep-space missions

**DOI:** 10.1093/jrr/rrt202

**Published:** 2014-03

**Authors:** Xinhua Yan, Sharath P. Sasi, Hannah Gee, Juyong Lee, Yongyao Yang, Jin Song, Joseph Carrozza, David A. Goukassian

**Affiliations:** 1 CardioVascular Research Center, GeneSys Research Institute, Boston, MA, USA; 2 Cardiovascular Division, Steward St Elizabeth's Medical Center, Boston, MA, USA; 3 Tufts University School of Medicine, Boston, MA, USA

**Keywords:** HZE, iron, proton, low-dose, cardiovascular risks, Ca^2+^

## Abstract

Background: During the future Moon and Mars missions, astronauts will be exposed to space radiation (IR) for extended time. The majority of space flight-associated risks identified for the cardiovascular (CV) system to date were determined shortly after low Earth orbit (LEO) short- and long-duration space flights that include: serious cardiac dysrhythmias, compromised orthostatic CV response and manifestation of previously asymptomatic CV disease. Further ground-based experiments using a surrogate model of microgravity supported the space flight data for significant cardiac remodeling due to prolonged exposure to microgravity. These symptoms were determined to be a consequence of adaptation to microgravity that could be ameliorated by a post-mission exercise program, and were not identified as risk factors that were causatively related to space IR. Long-term degenerative effects of cosmic IR during and after space flights on CV system are unknown.

It was suggested that due to GCR, each cell in an astronaut's body will be traversed by ^1^H every 3 days, helium (^2^He) nuclei every few weeks and high charge and energy (HZE) nuclei (e.g. ^28^Si, ^56^Fe) every few months. Despite the fact that only 1% of GCR is composed of ions heavier than helium, ∼41% of the IR dose-equivalent is predicted to be HZE particles with 13% being from ^56^Fe particles, only. During an exploration-class space mission to Mars, astronauts will not have access to comprehensive healthcare services for a period of at least 2–3 years. Since the majority of experienced astronauts are middle-aged (average age is 46, and the range is 33–58 years), they are at risk for developing serious CV events which could be life-threatening for the astronaut and mission-threatening for NASA. Therefore, it is important to evaluate the effects and potential CV risks caused by space IR. We hypothesized that: (i) low-dose space IR-induced biological responses may be long-lasting and are IR type-dependent; (ii) IR may increase CV risks in the aging heart (IR + AGING model) and affect the heart recovery after an adverse CV event, such as acute myocardial infarct (IR + AGING + AMI model).

Methods: Eight- to 9-month-old C57BL/6N male mice were IR once with proton (^1^H) 50 cGy, 1 GeV/n or iron (^56^Fe) 15 cGy, 1 GeV/n. We evaluated IR-induced biological tissue responses—underlying molecular mechanisms, calcium handling, signal transduction, gene expression and cardiac fibrosis. Cardiac function was assessed by echocardiography (ECHO) and hemodynamic measurements (HEMO) as detailed in Fig. 1. AMI was induced by ligation of left anterior descending coronary artery 1 and 3 months post-IR as detailed in Fig. 2.

**Fig. 1.**
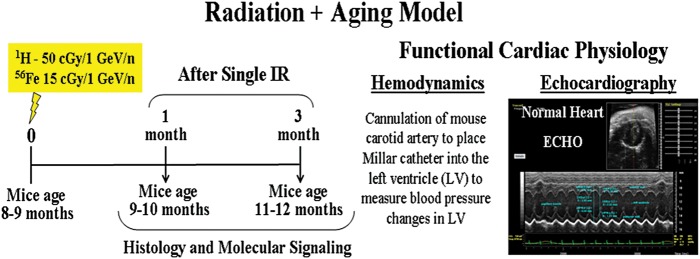
Radiation + aging model.

**Fig. 2.**
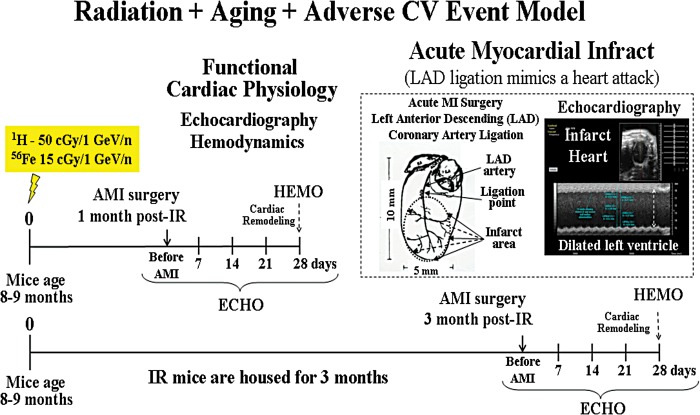
Radiation + aging model + adverse CV event model.

Radiation + aging model.

Radiation + aging model + adverse CV event model.

Results: In the IR + AGING model, cardiac function was not different among the control and ^1^H-IR group, whereas left ventricular end-diastolic pressure (LVEDP) was significantly increased in ^56^Fe mice 1 and 3 months post-IR. There was a small but statistically significant (*P* < 0.04) improvement of ejection fraction % (EF%) in ^1^H-IR vs control mice. One month post-IR, compared with control, ^1^H- and ^56^Fe-IR hearts had a significant up-regulation of sarcolemmal Na^+^–Ca^2+^ exchanger (NCX) (∼200% *P*<0.007), sarco(endo)plasmic reticulum calcium-ATPase (SERCA2a, >200% increases, *P* < 0.02) and 400% decreases in p-p38 MAPK (*P* < 0.05), suggesting activation of compensatory mechanisms in [Ca^2+^]_*i*_ handling in these hearts. By 3 months, compared with control, ^1^H- and ^56^Fe-IR hearts had 200–500% (*P* < 0.02) decreases in SERCA2a and more than 200% decreases in p-Creb-1 (*P* < 0.02), suggesting reduced capacity in intracellular [Ca^2+^]_*i*_ handling. These data suggest that dysfunction in [Ca^2+^]_*i*_ handling combined with LVEDP increase after ^56^Fe-IR may arise from the excessive demand on the heart due to prolonged activation of compensatory mechanisms that lead to changes in SERCA2a and p-Creb1 levels. This may represent a possible intracellular mechanism of heart failure in development in ^56^Fe-IR hearts.

In the IR + AGING + AMI model, no mortality was observed among three different groups 1 or 3 months post-IR and up to 28 days post-AMI. However, 1 month post-IR and 28 days post-AMI, the infarct size was significantly smaller in ^56^Fe-IR (p < 0.003) and ^1^H-IR (p = n.s.) vs control-IR mice, suggesting that at 1 month, ^56^Fe-IR primes the heart to recover better after AMI. In contrast, 3 months post AMI, ^1^H-AMI mice had a better cardiac functional recovery compared with control-AMI and ^56^Fe-AMI mice. The ejection fraction (EF%) was most decreased in ^56^Fe-AMI mice (^56^Fe-AMI vs ^1^H-AMI: 18 vs 48%, *P* < 0.007, ∼65–70% pre-AMI EF% for all groups). There was a 2- to 4-fold increase in LVEDP in ^56^Fe-AMI vs ^1^H-AMI (*P* < 0.04), suggesting that ^56^Fe-AMI hearts developed cardiac de-compensation. Western blots showed that 3 days post-AMI, compared with control- and ^1^H-IR-AMI mice, ^56^Fe-IR-AMI hearts had a 4- to 7-fold (*P* < 0.04) decreases in p-Akt (Thr308), p-Erk1/2 (*P* < 0.007) and ∼2-fold (*P* < 0.01) increase in phosphorylated ribosomal protein S6 kinase (p-S6k, a readout for mTORC1 pathway activation), suggesting decreased survival and angiogenesis signaling and decreased autophagy in these hearts. Seven days post-AMI, the levels of p-pErk1/2 were comparable between all three treatment conditions. However, in ^56^Fe-IR-AMI hearts, the p-Akt (Thr308) levels remained 4-fold decreased. Additionally, here was a 3-fold (*P*<0.05) decrease in p-S6k levels and >10-fold increase in p-p38 MAPK level in ^56^Fe vs control and ^1^H-IR-AMI hearts, suggesting continuous decreases in the survival, proliferation and angiogenesis signaling (p-Akt and p-S6k) and increase in the apoptotic signaling (p-p38 MAPK) up to Day 7 post-AMI in ^56^Fe-IR-AMI mice.

In summary, our results revealed that by 1 and 3 months post-IR in IR + AGING, ^56^Fe-IR but not ^1^H-IR mice had worse cardiac function. Further, a single ^1^H-IR 3 months prior to AMI improved, whereas ^56^Fe-IR worsened, recovery from AMI recovery. Our data in the IR + AGING and IR + AGING + AMI groups strongly suggest that low-dose HZE particle IR (^56^Fe) have long-lasting negative effect on heart homeostasis during normal aging, and present a significant CV risk for recovery after adverse CV event, such as AMI.

